# Supplementary material for: Dose-Dependent Beneficial Effects of Tryptophan and Its Derived Metabolites on Akkermansia In Vitro: A Preliminary Prospective Study
Source: Microorganisms. 2021 Jul 14;9(7):1511. doi: 10.3390/microorganisms9071511 (PMC8305782; doi:10.3390/microorganisms9071511)
Supplement: Supplementary file 1 [file microorganisms-09-01511-s001.zip › microorganisms-1287666-supplementary.pdf]

Supplementary materials

Dose-Dependent Beneficial Effects of Tryptophan and Its Derived Metabolites on *Akkermansia* in vitro: A Preliminary Prospective Study

Jia Yin, Yujie Song, Yaozhong Hu, Yuanyifei Wang, Bowei Zhang, Jin Wang, Xuemeng Ji, and Shuo Wang*

Tianjin Key Laboratory of Food Science and Health, School of Medicine, Nankai University, Tianjin 300071, China; spyinjia@163.com (J.Y.); yjsongqcyy@163.com (Y.S.); yzhu@nankai.edu.cn (Y.H.); wangyyf163@163.com (Y.W.); bwzhang@nankai.edu.cn (B.Z.); wangjin@nankai.edu.cn (J.W); jixuemeng@nankai.edu.cn (X.J.)

* Correspondence: wangshuo@nankai.edu.cn; Tel.: +86-22-85358445

Table S1. Operating parameters in MRM-mode.

Compound	Molecular formula	M (g/mol)	M+X+ (m/z)	Fragments (m/z)	DP (V)	EP (V)	CE (V)
				Q1 177.20 → 160.20	16	6	20
5-hydroxytryptamine (5-HT)	$C_{10}H_{12}N_2O$	176.21	$M + H^+$				
				Q2 177.20 → 132.00	16	8	30
				Q1 209.00 → 192.00	11	11	11
Kynurenine (Kyn)	$C_{10}H_{12}N_2O_3$	208.21	$M + H^+$	Q2 209.00 → 146.00	29	29	29
				Q3 209.00 → 94.00	25	25	25
				Q1 188.20 →	35	12	21

				146.10			
3-Indoleacrylic acid (IA)	$C_{11}H_9NO_2$	187.19	M + H^+	Q2 188.20 → 160.10	36	6	18
				Q3 188.20 → 170.20	16	9	18
				Q1 176.10 → 130.00	30	10	35
Indole acetate (IAA)	$C_{10}H_9NO_2$	175.18	M + H^+	Q2 176.10 → 103.10	30	10	35
				Q3 176.10 → 77.10	30	10	35
Indole-3-Acetamide (IAM)	$C_{10}H_{10}N_2O$	174.20	M + H^+	Q1 175.20 → 130.20	29	29	29
				Q2 175.20 → 105.00	30	30	30
Indole-3-carboxaldehyde (Icld)	C_9H_7NO	145.16	M + H^+	Q1 146.10 → 118.00	24	24	24
				Q2 146.10 → 118.00	35	35	35
Indole lactate (ILA)	$C_{11}H_{11}NO_3$	205.21	M + H^+	Q1 206.20 → 170.10	33	33	33
				Q2 206.20 →	33	33	33

				118.30			
Indole	C_8H_7N	117.15	$M + H^+$	Q1 118.00 → 91.00	9	10	30
				Q2 118.00 → 63.00	9	10	24
3-Indolepropionic acid (IPA)	$C_{11}H_{11}NO_2$	189.21	$M + H^+$	Q1 190.20 → 130.30	29	12	26
				Q2 190.20 → 172.30	25	8	21

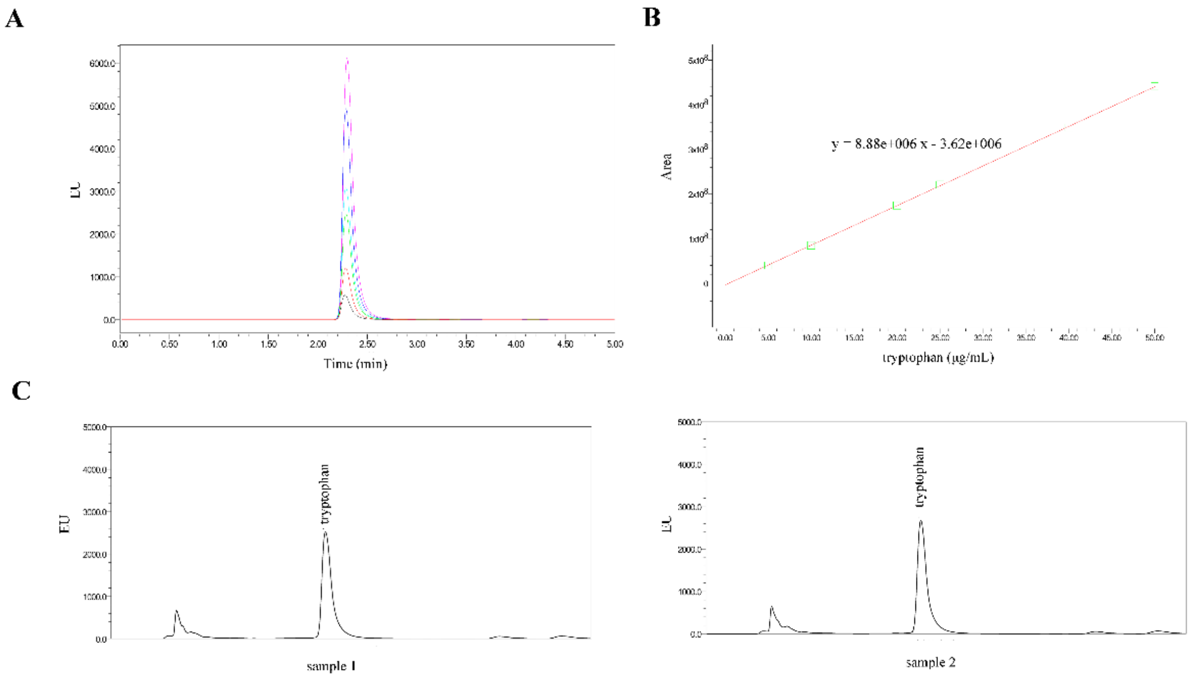

Figure S1. The analysis of the level of tryptophan in BHI medium. (A) Standards overlapping chromatogram; (B) Standard curve; (C) Analysis of tryptophan contents of BHI medium.

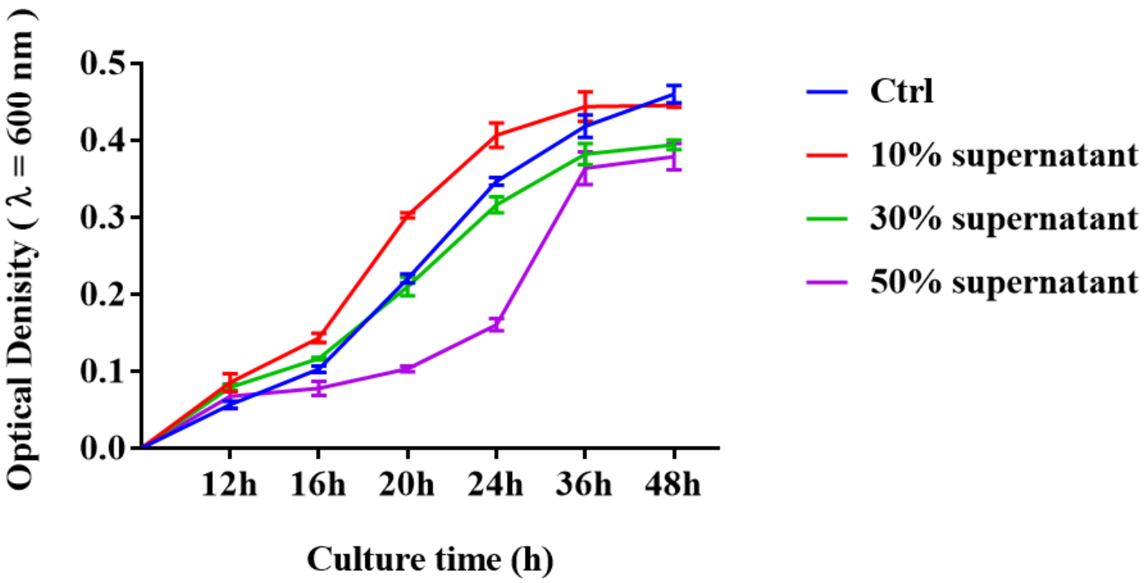

Figure S2. The OD600 of *A. muciniphila* BAA-835^{T} cultured with supernatant of *A. muciniphila* BAA-835^{T}.
